# Simple and large-scale chromosomal engineering of mouse zygotes via *in vitro* and *in vivo* electroporation

**DOI:** 10.1038/s41598-019-50900-y

**Published:** 2019-10-11

**Authors:** Satoru Iwata, Hitomi Nakadai, Daisuke Fukushi, Mami Jose, Miki Nagahara, Takashi Iwamoto

**Affiliations:** 1 Center for Education in Laboratory Animal Research, Chubu University, Kasugai, Japan; 2 Department of Biomedical Sciences, College of Life and Health Sciences, Chubu University, Kasugai, Japan; 3 College of Bioscience and Biotechnology, Chubu University, Kasugai, Japan; 4 Department of Genetics, Institute for Developmental Research, Aichi Developmental Disability Center, Kasugai, Japan

**Keywords:** Genetic engineering, Genetic engineering

## Abstract

The clustered regularly interspaced short palindromic repeats (CRISPR)/Cas9 system has facilitated dramatic progress in the field of genome engineering. Whilst microinjection of the Cas9 protein and a single guide RNA (sgRNA) into mouse zygotes is a widespread method for producing genetically engineered mice, *in vitro* and *in vivo* electroporation (which are much more convenient strategies) have recently been developed. However, it remains unknown whether these electroporation methods are able to manipulate genomes at the chromosome level. In the present study, we used these techniques to introduce chromosomal inversions of several megabases (Mb) in length in mouse zygotes. Using *in vitro* electroporation, we successfully introduced a 7.67 Mb inversion, which is longer than any previously reported inversion produced using microinjection-based methods. Additionally, using *in vivo* electroporation, we also introduced a long chromosomal inversion by targeting an allele in F_1_ hybrid mice. To our knowledge, the present study is the first report of target-specific chromosomal inversions in mammalian zygotes using electroporation.

## Introduction

Chromosomal rearrangements are the cause of many hereditary disorders and play a role in the pathogenesis of diseases such as cancer^1,2^. To study these molecular pathologies, *in vivo* models of chromosomal rearrangement are indispensable, but producing them requires substantial effort and prudent technique^3–6^. The clustered regularly interspaced short palindromic repeat (CRISPR)/CRISPR-associated (Cas) system has enabled genome editing of specific sites and has been applied for the generation of gene knockouts and knockins in rats, mice and other organisms^7^. Recently, chromosomal rearrangements, including large deletions, inversions and duplications, were also performed via microinjection of Cas9 and single guide RNAs (sgRNAs) into mouse zygotes^8,9^. However, this method still requires specialised equipment and highly skilled personnel. To overcome this limitation, various groups have reported successful genome editing via the *in vitro* electroporation of Cas9 and a sgRNA into mouse zygotes^10–13^, which greatly simplifies their delivery and significantly improves embryo/pup survival compared to microinjection-based methods^11,12^. More recently, Ohtsuka *et al*. developed a novel genome editing method called improved genome editing via oviductal nucleic acid delivery (*i*-GONAD)^14,15^, which directly delivers Cas9 and a sgRNA into rodent zygotes through the oviducts^14,15^. This method does not require the isolation, culture, transfer or other *in vitro* handling of embryos. Whereas this approach has been applied for making knockout and knockin rodents, it is unknown whether it is able to generate more drastic chromosomal rearrangements, such as inversions. In the present study, we present an editing method to generate chromosomal rearrangements of substantial size using *in vitro* and *in vivo* electroporation in mouse zygotes. The inverted region in our study was 7.67 megabases (Mb), which is approximately 1.5 times longer than a previously reported inversion produced in mouse zygotes^8^. Moreover, we demonstrate a method for targeting a selected allele in F_1_ hybrid mice using *in vivo* electroporation. This method resulted in successful genomic rearrangements, generating large-scale inversions and recessive lethal deletion alleles at specific sites. Our study shows that the application of the CRISPR/Cas9 system via electroporation *in vitro* and *in vivo* would be useful for the study of genetic diseases with large-scale chromosomal rearrangements.

## Results and Discussion

### Chromosomal inversion via *in vitro* electroporation

**Figure 1:**
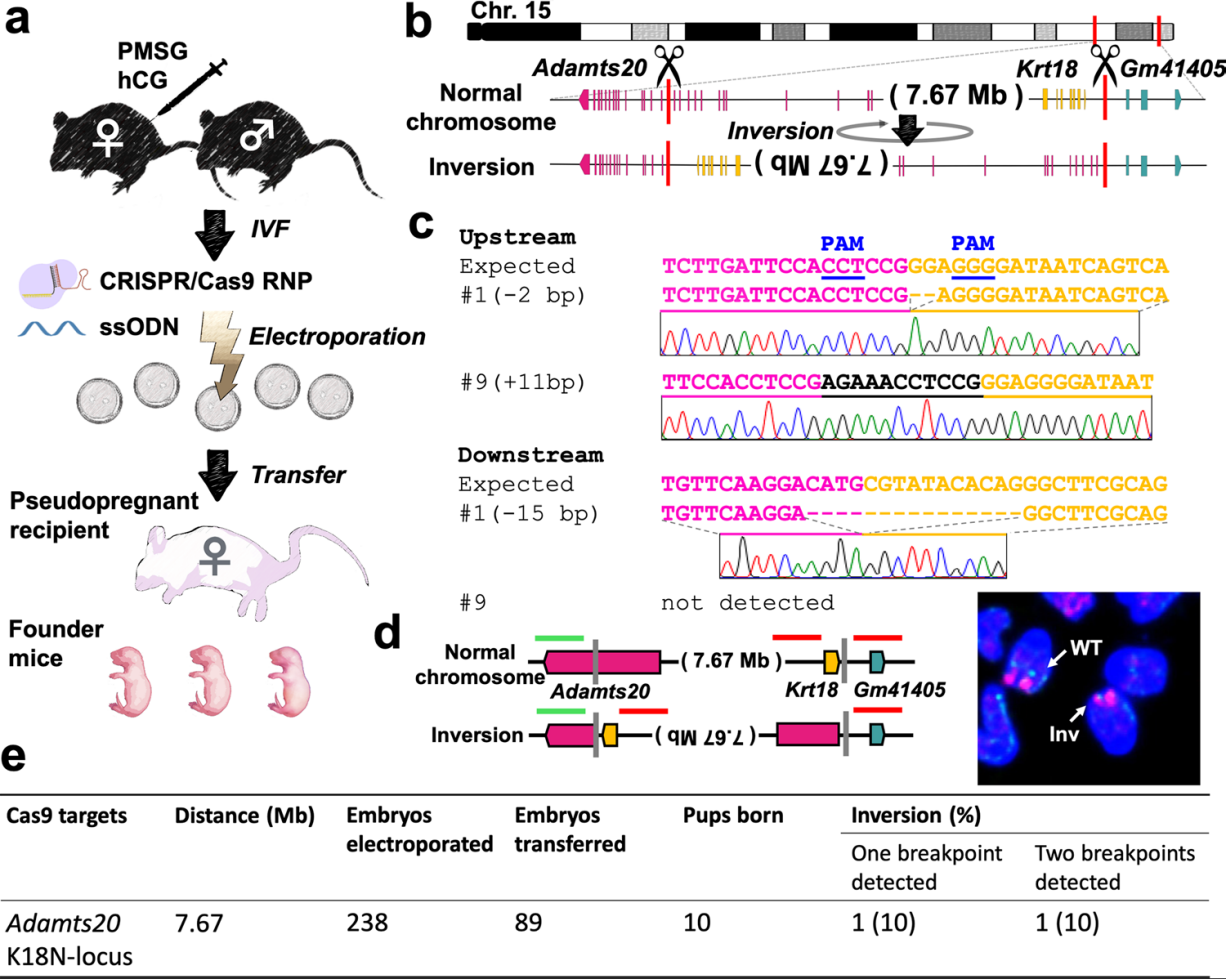
Chromosomal engineering of mouse zygotes via *in vitro* electroporation. (**a**) Experimental procedures for induction of chromosomal inversion using *in vitro* electroporation. (**b**) Schematic of the chromosomal rearrangements created by an inversion between *Adamts20* and the K18N-locus in chromosome 15. (**c**) Alignment of sequences corresponding to the *Adamts20* and the K18N-locus genomic breakpoint junctions. (**d**) Schematic of an inversion event and the associated FISH signal pattern. Wild-type (WT) shows a separation of the green and red signals. Inversion (Inv) shows the co-localization of the signals. (**e**) Summary of the experimental efficiency of chromosomal inversions introduced via *in vitro* electroporation.

We examined whether *in vitro* electroporation-mediated CRISPR/Cas9 could generate large-scale genome editing, such as megabase-sized chromosomal inversion. The CRISPR/Cas9 mixture was electroporated into mouse zygotes as described in the Methods section (Fig. 1a, Supplementary Fig. 1a,b). We first designed a 7.67 Mb inversion between *Adamts20* and the *Krt18* neighbourhood locus (K18N-locus) on the right arm of chromosome 15 (Fig. 1b), which includes the largest region of the type II keratin gene cluster; we targeted this region because disruption of *Adamts20* would show easily identifiable phenotypes^16^. In the present study, we used the cloning-free CRISPR/Cas system, consisting of Cas9 protein, chemically synthesised CRISPR RNA (crRNA), and trans-activating crRNA (tracrRNA). In addition, we designed two single-stranded oligodeoxynucleotides (ssODNs) that joined the chromosomal breakpoints together; each ssODN had a 100 bp sequence that was homologous to each junction point so that chromosomal inversion would be induced by the homology-directed repair (HDR) process to occur between the targeted regions and the homologous ssODNs (Supplementary Fig. 2).

We electroporated CRISPR/Cas9 ribonucleoproteins (RNPs) targeting *Adamts20*, the K18N-locus, and the ssODNs into *in vitro* fertilised C57BL/6JJmsSlc zygotes. Embryos were allowed to develop to the two-cell stage, and then they were transferred into pseudopregnant Slc:ICR mice. Six of the 12 Slc:ICR foster mothers bore pups through caesarean section, some of which showed the white-spotted phenotype (Supplementary Fig. 3). We screened for F_0_ mice using PCR amplification and DNA sequencing of both junction points, and we detected chromosomal inversion in one of the 10 F_0_ mice (10%) (Fig. 1c,e). However, in one possible inversion mouse line, named #9, only one junction point was detected, similar to what has been previously reported^8^; this result suggests that one or both of the primer binding sites may have been deleted (Fig. 1c). To confirm the inversion of line #9, we performed fluorescence *in situ* hybridization (FISH). Whereas the normal chromosome showed discrete green and red signals from the *Adamts20* and K18N loci, respectively, overlapping signals indicative of the inverted *Adamts20*-K18N locus were observed in the chromosome of line #9, suggesting that line #9 contained the 7.67 Mb inversion (Fig. 1d). Previous studies have demonstrated that recombination between the wild-type and chromosomal inversion line does not occur within these inversion events^17–19^ (Supplementary Fig. 4a). To determine whether crossovers were suppressed in mouse line #9, heterozygous #9 (C57BL/6JJmsSlc background) and C57BL/6JJmsSlc females were mated with C3H/HeJYokSlc males, and the F_1_ heterozygotes were further backcrossed to C3H/HeJYokSlc mice. Among the 18 mice harbouring heterozygous inversions that were examined, there was no recombination detected within this inversion. In contrast, the mice with homozygous wild-type chromosomes did not show these suppressive effects, supporting the idea that inversion-suppressed recombination was present in line #9 (Supplementary Fig. 4b,c). These results demonstrate that our electroporation method introduced a 7.67 Mb chromosomal inversion in the mouse zygotes, which is 1.5 times longer than the inversion produced in a previous study using microinjection-based methods^8^. Moreover, the efficiency of the inversion produced using electroporation was comparable to that achieved using the microinjection method^8,9^. However, survival rates were low: only 37.4% (89/238) at the two-cell stage. This may be due to the embryonic lethality of homologous inversion in these loci, although further investigation is needed.

### Chromosomal inversion via *in vivo* electroporation

**Figure 2:**
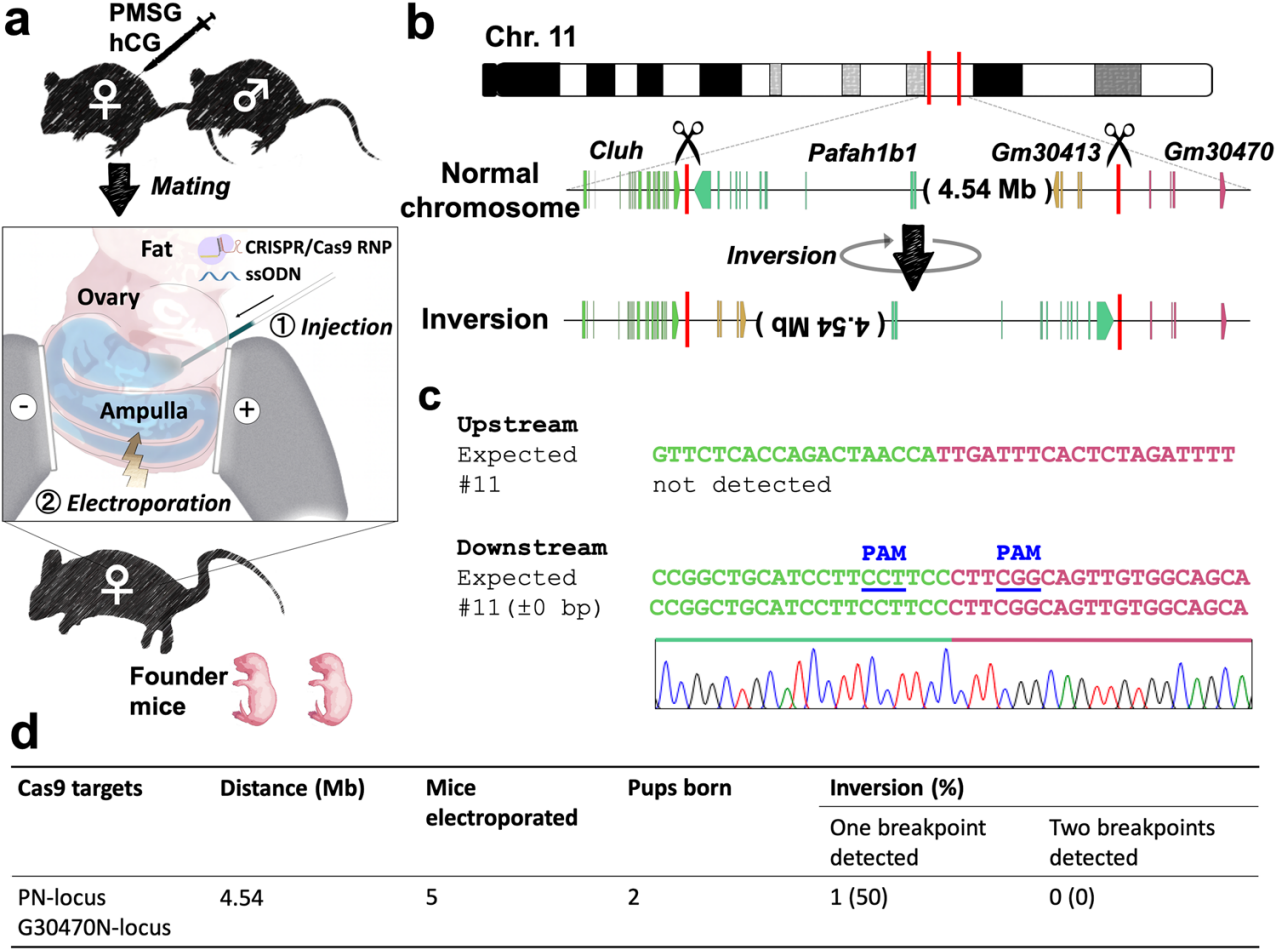
Chromosomal engineering of mouse zygotes via *in vivo* electroporation. (**a**) Experimental procedures for induction of chromosomal inversion using *in vivo* electroporation. (**b**) Schematic of the chromosomal rearrangements created by an inversion between the PN-locus and G30470-locus in chromosome 11. (**c**) Alignment of the sequences corresponding to the PN-locus and the G30470-locus genomic breakpoint junctions. (**d**) Summary of the experimental efficiency of chromosomal inversion via *in vivo* electroporation.

We attempted to generate a chromosomal inversion via a previously developed *in vivo* electroporation technique called improved genome editing via oviductal nucleic acid delivery (*i*-GONAD)^14,15^ (Fig. 2a, Supplementary Fig. 1a,c,d). This method can bypass the following three steps: (1) zygote isolation, (2) microinjection, and (3) zygote transfer^14^. We attempted to introduce a 4.54 Mb inversion between the *Pafah1b1* neighbourhood locus (PN-locus) and the *Gm30470* neighbourhood locus (G30470N-locus) in the middle of chromosome 11 (Fig. 2b); we selected this site because the disruption of neither of the two target regions would produce a lethal phenotype. We injected the CRISPR/Cas9 RNPs targeting the PN-locus and G30470N-locus and the ssODNs into the oviduct lumen of five superovulated pregnant C57BL/6NCrSlc females at day 0.7 of pregnancy (corresponding to the late one-cell stage) and electroporated the oviduct *in vivo*. Only one female became pregnant and two F_0_ pups were obtained through caesarean section, and one pup had a chromosomal inversion (#11) (Fig. 2c,d), with one complete copy of the ssODN at one breakpoint. However, another breakpoint was unmapped, suggesting that mouse line #11 may have had an inversion with a deletion of the primer binding sites, similar to what was observed in line #9 (Fig. 2c). Whilst the efficiency is low, these results suggest that *in vivo* electroporation could be used to introduce a megabase-sized chromosomal inversion.

### Allele-specific inversion in F_1_ hybrid mice via *in vivo* electroporation

Whereas a previous study of *in vivo* electroporation genome editing in mouse zygotes reported an average litter size of approximately 5^14^, only two pups were obtained in our experiment.

**Figure 3:**
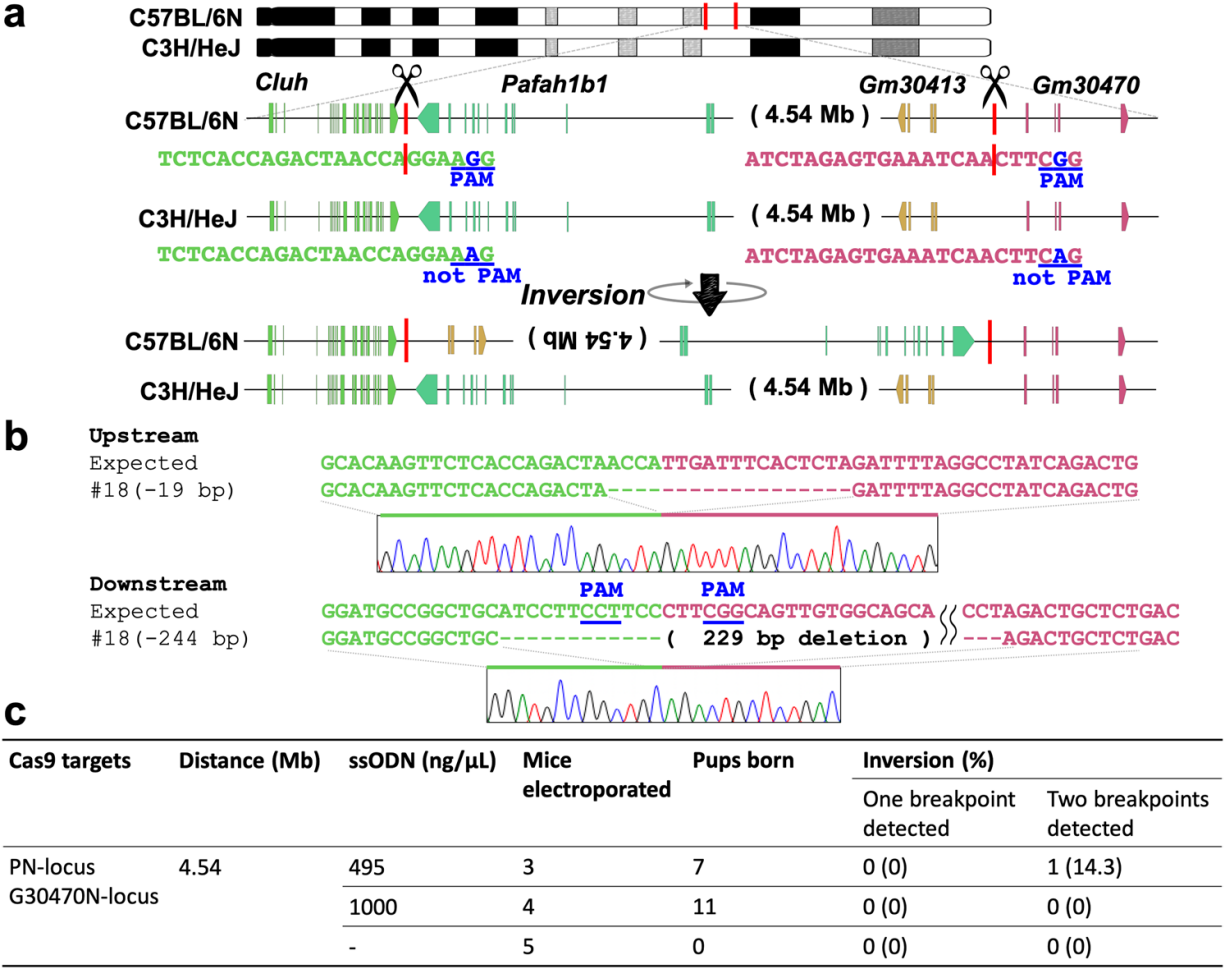
Chromosomal engineering of mouse zygotes via targeting the selected allele in F_1_ hybrid mice using *in vivo* electroporation. (**a**) Schematic of the chromosomal rearrangements in F_1_ hybrid mice. The rearrangements were created via an inversion between the PN-locus and G30470-locus in only the C57BL/6NCrSlc allele. (**b**) Alignment of sequences corresponding to the PN-locus and G30470-locus genomic breakpoint junctions. (**c**) Summary of the experimental efficiency of chromosomal inversion via *in vivo* electroporation.

As the efficiency of the CRISPR/Cas system is very high, isolation of chromosomal rearrangement in mouse zygotes is sometimes difficult using this system because of the frequent deletion of both copies of the target regions, which results in lethality^20^. Hence, the low efficiency in our *in vivo* experiments may result in part from the biallelic megabase-sized deletion. To exclude this possibility, we developed a unique *in vivo* electroporation method wherein one allele was selectively edited in F_1_ hybrid (B6C3F1) mice (Fig. 3a). Using the Mouse Phenome Database (https://phenome.jax.org/), we selected two SNPs; both existed in the protospacer adjacent motif (PAM) sequences of the C57BL/6NCrSlc genome, but they are absent in the PAM sequences of the C3H/HeJYokeSlc genome. We performed *in vivo* electroporation on three C57BL/6NCrSlc females that had been mated to C3H/HeJYokSlc males, and we used two sgRNAs that selectively targeted the C57BL/6NCrSlc genome. We obtained 7 F_0_ pups through caesarean section and successfully obtained one mouse in which an inversion at both junction points was detected (Fig. 3b,c). Additionally, we examined 11 blastocysts from the pregnant female mice three days after the *in vivo* electroporation procedure and found that one embryo contained the inversion at both break points (Supplementary Fig. 5). To improve the low inversion efficiency, we tried the experiment again with approximately double the concentration of donor ssODN (1000 ng/µL) but could not find any breakpoints in 11 pups (Fig. 3c). In contrast, we reduced the concentration of ssODN to 250 ng/μL and analysed the results at the blastocyst stage. As shown in Supplementary Fig. 5, we detected one breakpoint in two of 16 embryos (12.5%), but we did not detect two breakpoints in any of them. No pups were delivered in the absence of ssODN (Fig. 3c).

Together, our results indicate that genome editing via ssODN-dependent *in vivo* electroporation could elicit chromosomal inversion in mouse embryos, although further improvement of the method is required to increase the efficiency.

Upon closer inspection of the repaired regions in the inversions of thirteen mice (#1, #9, #11, #18, #23, #24, #27, #28, #29, #33, #32, #47, #48) obtained using the ssODNs, we found that only two mice (#11, #48) contained the complete sequence of the ssODN at one breakpoint (Fig. 2c, Supplementary Fig. 5b). Three mice (#1, #18, #32) had indels at both breakpoints (Figs 1c, 3b, Supplementary Fig. 5b), and seven (#9, #23, #24, #27, #28, #29, #30, #47) had indels at one breakpoint (Fig. 1c, Supplementary Fig. 5b). Interestingly, we observed that the upstream inversion junctions in seven mice had deletions near a 3-bp microhomology sequence of ‘CTA’ (#18, #23, #24, #27, #28, #29, #32) (Fig. 3b, Supplementary Fig. 5b). Consistent with a previous report, this observation suggests that a breakpoint flanked by microhomology sequences can generate inversion events via microhomology-mediated repair^21^. Thus, of the thirteen breakpoints examined, two appeared to be repaired by HDR, whereas eleven were likely to be repaired by microhomology-mediated end joining (MMEJ) or nonhomologous end joining (NHEJ).

### Allele-specific recessive lethal deletion in F_1_ hybrid mice via *in vivo* electroporation

**Figure 4:**
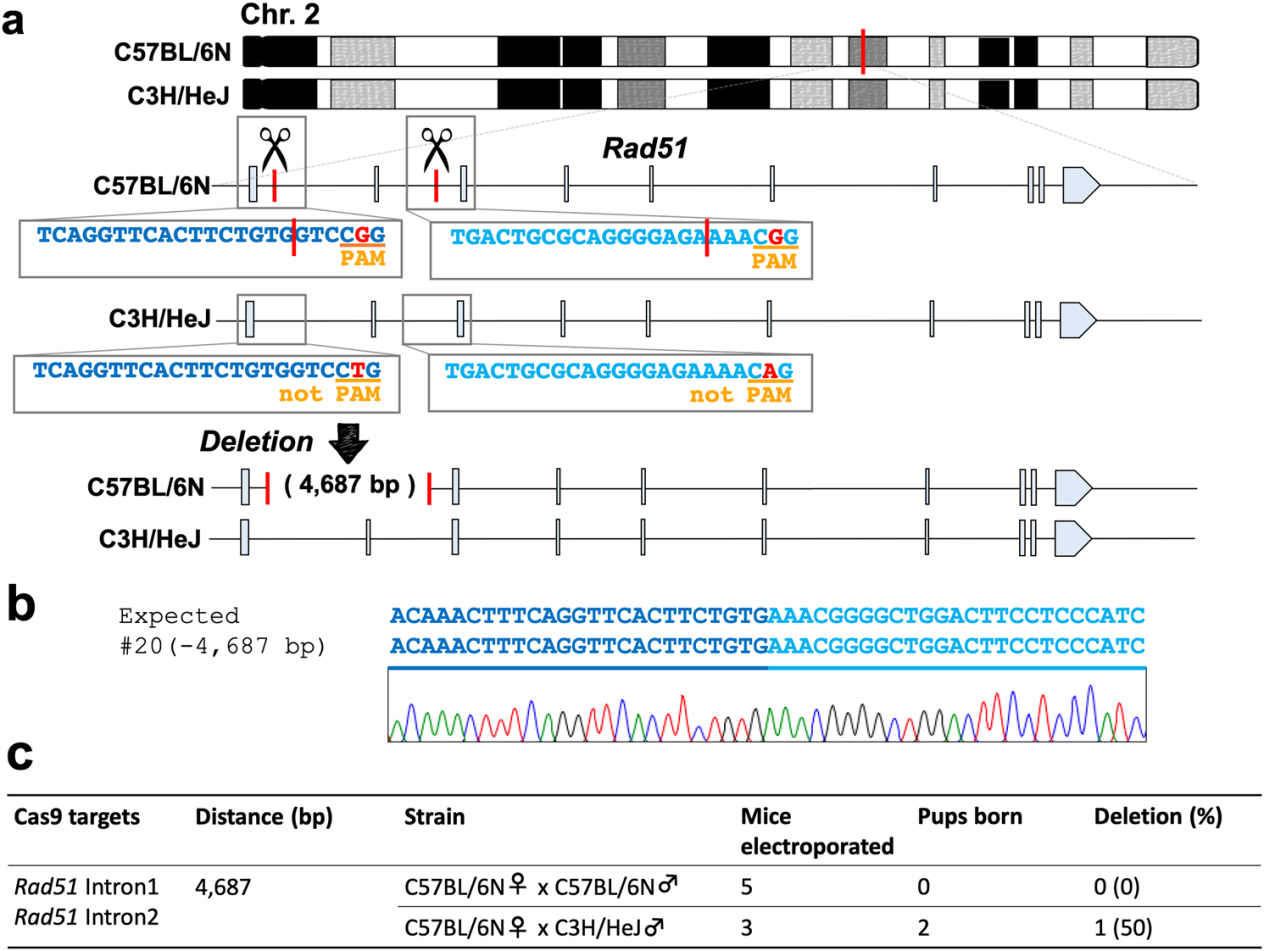
Lethal gene deletion in mouse zygotes via targeting the selected allele in F_1_ hybrid mice using *in vivo* electroporation. (**a**) Schematic of *Rad51* gene deletion in F_1_ hybrid mice, which was generated in only the C57BL/6NCrSlc allele. (**b**) Alignment of sequences corresponding to the *Rad51* intron 1 and intron 2 genomic breakpoint junctions. (**c**) Summary of the experimental efficiency of chromosomal deletion via *in vivo* electroporation.

Next, we examined whether this strategy could detect a recessive lethal deletion (Fig. 4a). We attempted to delete an essential gene, *Rad51*, the loss of which in both alleles seemed to produce a lethal embryonic phenotype^22,23^. We electroporated the genome editing CRISPR/Cas9 mixture into the oviduct of C57BL/6NCrSlc females that had been mated to C3H/HeJYokSlc males, and we used C57BL/6NCrSlc females mated to C57BL/6NCrSlc males as a control. In the C57BL/6NCrSlc/C3H/HeJYokSlc F_1_ hybrid strains, we obtained two F_0_ pups and found that one of them had a large deletion in the target locus (Fig. 4b,c). It should be noted that the control C57BL/6NCrSlc/C57BL/6NCrSlc strain failed to deliver their pups, suggesting that these embryos died because of the deletion of both *Rad51* gene copies (Fig. 4c). In fact, the deletion line #20 generated exhibited a recessive lethal embryonic phenotype (Supplementary Fig. 6). Together, our results suggest that this unique method may be successfully applied for the generation of target-specific chromosomal rearrangements, such as large-scale inversions and recessive lethal deletions.

## Conclusions

To our knowledge, this is the first study to successfully introduce chromosomal inversions in mouse zygotes using electroporation-based methods. Moreover, *in vivo* allele-specific genome editing in F_1_ hybrid mice has allowed us to efficiently introduce chromosomal inversions and recessive lethal deletions. Together, our methods provide a simple and efficient approach for engineering chromosomal rearrangements and contribute to the study of disease caused by chromosomal rearrangements.

## Methods

### Animal strains

C57BL/6JJmsSlc, C57BL/6NCrSlc, C3H/HeJYokSlc, and Slc:ICR mice (8–12 weeks old) were obtained from Japan SLC (Shizuoka, Japan). The animals were kept at a constant temperature (22 °C ± 2 °C) and humidity (50% ± 10%), with a 12 h light/12 h dark cycle. All animal experiments were approved by the Institutional Animal Care and Use Committee of Chubu University (Permit Numbers #2910066, #2910067 at Chubu University) and were conducted in accordance with institutional guidelines.

### CRISPR RNP and ssODN preparation

The CRISPR guide RNAs were designed using CHOPCHOP (http://chopchop.cbu.uib.no/)^24^ (Supplementary Table 1). To avoid possible off-target events, we selected gRNAs that are as specific for the target sequence as possible. No mutations were detected using the T7 endonuclease I (T7E1) (New England BioLabs, MA, USA) assay at four off-target sites that differed by one or two base-pairs from the on-target sites (Supplementary Fig. 7). The T7E1 assay was performed as previously described^25^. The CRISPR RNP consists of Alt-R S.p. Cas9 Nuclease 3NLS (Integrated DNA Technologies, IL, USA) and a crRNA:tracrRNA duplex, which included the custom guide RNA (crRNA) and a universal structural RNA (tracrRNA) (Integrated DNA Technologies). The crRNA and tracrRNA were heated to 95 °C for 5 min and slowly cooled to room temperature. This crRNA:tracrRNA duplex and the Alt-R S.p. Cas9 Nuclease 3NLS were incubated at room temperature for 10 min to form the RNP complex.

The ssODNs were manufactured by Eurofins Genomics (Tokyo, Japan) and were designed to join two DNA sequences so that the junction would be positioned at the centre of the predicted cleavage sites, which were located within 3 bp of the PAM sequences (Supplementary Table 2).

### *In vitro* electroporation

C57BL/6JJmsSlc female mice were injected intraperitoneally with 7.5 IU PMSG (ASKA Animal Health., Tokyo, Japan), followed by 7.5 IU of hCG (ASKA Animal Health., Tokyo, Japan) 48 h later. Thirteen hours after the hCG injection, superovulated female mice were euthanised via cervical dislocation, and unfertilised oocytes isolated from the female mice were subjected to *in vitro* fertilisation (IVF) with freshly isolated spermatozoa from euthanised C57BL/6JJmsSlc male mice, as previously described^26^. To generate chromosomes with an inversion, the following concentrations of CRISPR reagents were used: 200 ng/μL Cas9 protein, 100 ng/μL upstream crRNA/tracrRNA, 100 ng/μL downstream crRNA/tracrRNA, 100 ng/μL upstream ssODN and 100 ng/μL downstream ssODN diluted in Opti-MEM (Thermo Fisher Scientific, MA, USA). The *in vitro* electroporation procedures were performed as previously described^10^. Briefly, the embryos were cultured in KSOM medium, washed with Opti-MEM, and then placed in an electrode cuvette (CUY505P5 [NEPA GENE., Chiba, Japan]) with CRISPR solutions (47 μL total volume), followed by electroporation using a NEPA21 (NEPA GENE). The following parameters were used for electroporation: poring pulse (voltage: 225 V; pulse length: 2.0 msec; pulse interval: 50 msec; number of pulses: 4; decay rate: 40%; polarity: +), transfer pulse (voltage: 20 V; pulse length: 50 msec; pulse interval: 50 msec; number of pulses: 5; decay rate: 40%; polarity: ±). After electroporation, the embryos were cultured to the two-cell stage in KSOM medium (Kyudo, Saga, Japan). Before embryo transfer, pseudopregnant Slc:ICR mice were anaesthetised with a mixture of three drugs: medetomidine (0.75 mg/kg), midazolam (4 mg/kg), and butorphanol (5 mg/kg). Following the embryo transfer, we placed the oviducts back in their original location and sutured the incisions. After the operation, atipamezole hydrochloride (0.3 mg/kg) was intraperitoneally injected to reverse the effects of the medetomidine.

### *In vivo* electroporation

For superovulation, C57BL/6NCrSlc female mice were injected intraperitoneally with 2 IU PMSG, and then they were injected with 5 IU hCG 48 h later, as previously described^27^. They were subsequently mated to C57BL/6NCrSlc or C3H/HeJYokSlc males. The presence of copulation plugs was confirmed via visual inspection the next morning, and plug-positive mice were subjected to *in vivo* electroporation experiments. To generate chromosomes with an inversion, the following concentrations of CRISPR solutions were used: 540 ng/μL Cas9 protein, 33 μM upstream and downstream crRNA/tracrRNA, 250, 495 or 1000 ng/μL upstream and downstream ssODN, and 0.05% Fast Green FCF (Wako) marker diluted in Opti-MEM (Thermo Fisher Scientific). To generate a chromosomal deletion, the following concentrations of CRISPR solutions were used: 500 ng/μL Cas9 protein, 30 μM upstream crRNA/tracrRNA, and 30 μM downstream crRNA/tracrRNA diluted in Opti-MEM (Thermo Fisher Scientific). Before *in vivo* electroporation, the females were anaesthetised with a mixture of three drugs: medetomidine (0.75 mg/kg), midazolam (4 mg/kg) and butorphanol (5 mg/kg). The CRISPR mixture (1 μL) was injected into the oviductal lumen upstream of the ampulla with a glass micropipette, which was made using a vertical capillary puller (NARISHIGE, Tokyo, Japan). After injecting CRISPR solutions, the oviduct regions were grasped by tweezer electrodes (CUY652P2.5 × 4 [NEPA GENE]), and electroporation was performed as previously described^14^ using a NEPA21 (NEPA GENE). The following parameters were used for electroporation: poring pulse (voltage: 50 V; pulse length: 5.0 msec; pulse interval: 50 msec; number of pulses: 3; decay rate: 10%; polarity: ±), transfer pulse (voltage: 10 V; pulse length: 50 msec; pulse interval: 50 msec; number of pulses: 3; decay rate: 40%; polarity: ±). After electroporation, we placed the oviducts back in their original location and sutured the incisions. After the operation, atipamezole hydrochloride (0.75 mg/kg) was intraperitoneally injected to reverse the effects of medetomidine.

### Analysis of the chromosome-engineered mice

To screen for chromosomal rearrangements, genomic DNA was isolated from the tails or ears of founder mice using lysis buffer (100 mM NaCl, 200 mM sucrose, 10 mM EDTA, 300 mM Tris (pH 8.0), and 1% SDS), and the DNA was examined via PCR amplification (Supplementary Figs 6a, 8 and Supplementary Table 3). PCR products were cloned into the pTAC-1 vector (Biodynamics, Tokyo, Japan), and sequences of individual clones were determined via Sanger sequencing (Eurofins Genomics).

### DNA extraction from mouse blastocysts

Crude DNA derived from each blastocyst was prepared to be a PCR template according to the method described^28^. Briefly, we euthanised electroporated females on embryonic day 3, flushed the uterine horn with 0.5 mL of M2 medium, collected blastocysts in a petri dish, and transferred a single blastocyst to a 0.2 ml PCR tube using a glass micropipette. Then, 10 μL of lysis buffer (50 mM KCl, 10 mM Tris-HCl (pH 8.3), 2.5 mM MgCl_2_, 0.1 mg/mL gelatin, 0.45% NP40, 0.45% Tween 20 and 125 μg/mL proteinase K) was added directly to each tube, and the samples were incubated at 56 °C for 30 min and then at 95 °C for 10 min. The target genomic regions were amplified by nested PCR with the primers listed in Supplementary Table 3.

### Cytogenetic analysis

Chromosomal samples were prepared from bone marrow cells of mouse line #9. The mice were injected intraperitoneally with 0.1 mg/μL colcemid and euthanised approximately 60 min afterwards. Chromosome isolation and FISH were performed as described with modification^29,30^. Each DNA probe, Adamts20 (15qE3), K18N (15qF2), and G41405N (15qF2), was amplified from the genomic DNA of a C57BL/6NCrSlc mouse by PCR (Supplementary Table 3). Adamts20 (15qE3) was labelled with Biotin-16-dUTP (Roche, Mannheim, Germany), and K18N (15qF2) and G41405N (15qF2) were labelled with Digoxigenin-11-dUTP (Roche), respectively. DNA probes were detected with Streptavidin Alexa Fluor® 488 conjugate (Thermo Fisher Scientific) or anti-digoxigenin-Rhodamine (Roche). Finally, the chromosomes were stained with 4′,6-diamidino-2-phenylindole (DAPI) (Roche).

### Test for recombination suppression

To examine whether recombination was suppressed in mouse line #9, heterozygous #9 (C57BL/6JJmsSlc background) females and C57BL/6JJmsSlc females were mated with C3H/HeJYokSlc males, and the F_1_ heterozygotes were further backcrossed to C3H/HeJYokSlc mice. The *Arf3* mutation (rs245124066) genotype of each individual was determined using PCR-restriction fragment length polymorphism analysis. Following PCR amplification, the PCR products were digested for 4 h at 37 °C with 5 units of StuI restriction enzyme, and they were then analysed via 1.2% agarose gel electrophoresis. Digestion of the PCR products with StuI yielded fragments of 269 and 146 bp in the C57BL/6JJmsSlc mice (*Arf3* (+/+)) vs. a single product of 415 bp in the C3H/HeJYokSlc mice (*Arf3* (−/−)). An *Arf3* (+/−) heterozygote yielded bands of 415 bp, 269 bp and 146 bp.

## Supplementary information

SUPPLEMENTARY INFORMATION
