# Supplementary material for: Simple and large-scale chromosomal engineering of mouse zygotes via in vitro and in vivo electroporation
Source: Sci Rep. 2019 Oct 11;9:14713. doi: 10.1038/s41598-019-50900-y (PMC6789149; doi:10.1038/s41598-019-50900-y)
Supplement: Supplementary file 1 — SUPPLEMENTARY INFORMATION [file 41598_2019_50900_MOESM1_ESM.pdf]

SUPPLEMENTARY INFORMATION

Simple and large-scale chromosomal engineering of mouse zygotes via *in vitro* and *in vivo* electroporation

Satoru Iwata[1,2,3,#], Hitomi Nakadai[2], Daisuke Fukushi[4], Mami Jose[2], Miki Nagahara[1], Takashi Iwamoto[1,2]

[1]Center for Education in Laboratory Animal Research, Chubu University
[2]Department of Biomedical Sciences, College of Life and Health Sciences, Chubu University
[3]College of Bioscience and Biotechnology, Chubu University
[4]Department of Genetics, Institute for Developmental Research, Aichi Developmental Disability Center
[#]To whom correspondence should be addressed: satoru_iwata@isc.chubu.ac.jp

Supplementary Figures:

Supplementary Fig. 1; Equipment for *in vitro* and *in vivo* electroporation of mouse zygotes.
Supplementary Fig. 2; Schematic design for creating chromosomal inversion using the CRISPR/Cas9 system.
Supplementary Fig. 3; Production of the chromosome-rearranged mouse via *in vitro* electroporation.
Supplementary Fig. 4; Chromosomal inversion #9 can suppress recombination.
Supplementary Fig. 5; Overview of blastocyst-stage embryo collection after *in vivo* electroporation and the sequences of the inversion junctions.
Supplementary Fig. 6; Genotype distribution in offspring from intercrossed heterozygous *Rad51* mice.
Supplementary Fig. 7; Off-target analysis by the T7E1 assay in the chromosome-rearranged mice.
Supplementary Fig. 8; Schematic representation of the PCR primer positions for the screening of chromosomal rearrangements.

Supplementary Tables:

Supplementary Table 1; List of gRNAs used in the present study
Supplementary Table 2; List of ssODNs used in the present study
Supplementary Table 3; List of all primers used in the present study

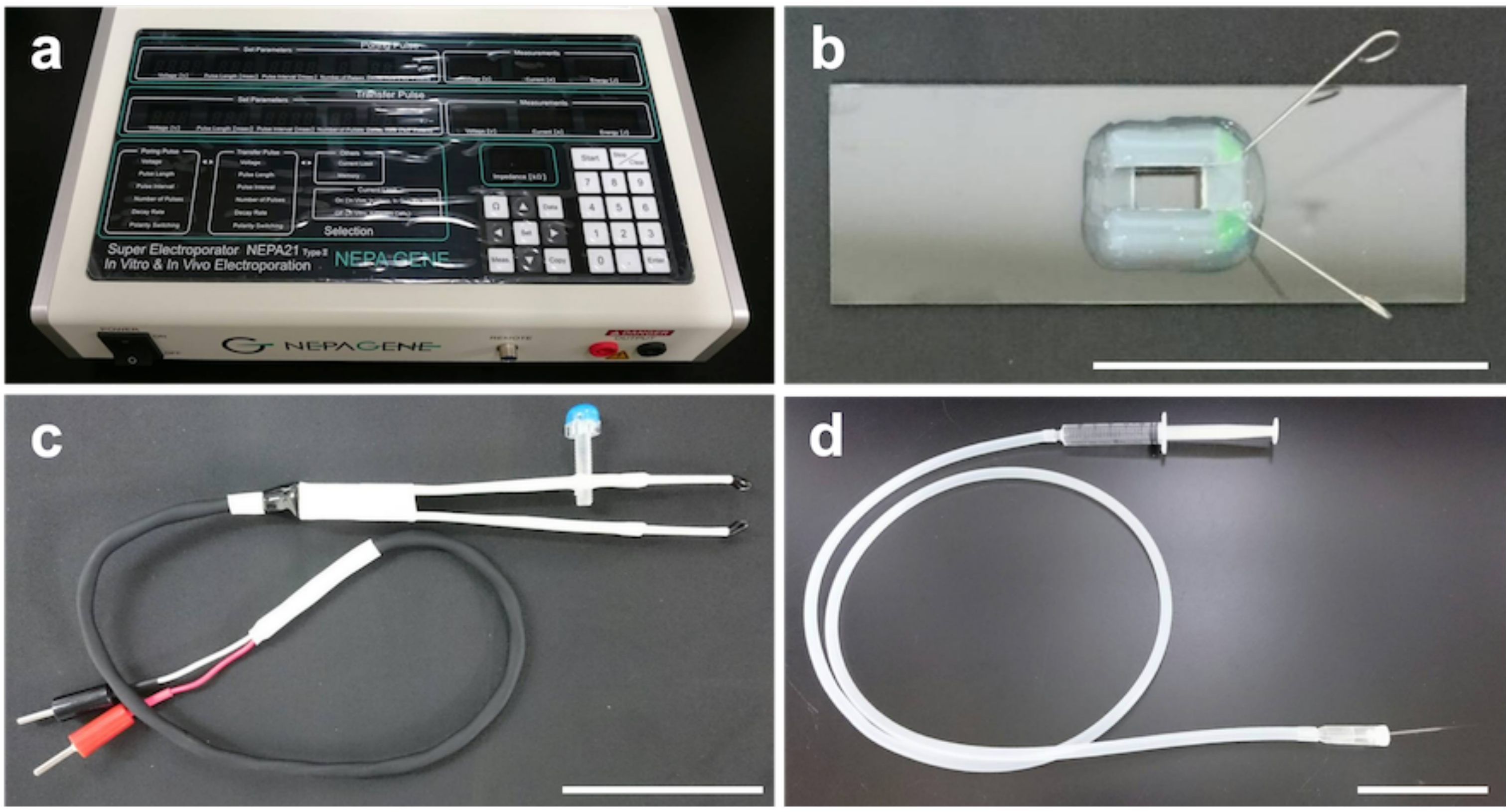

Supplementary Fig. 1: Equipment for *in vitro* and *in vivo* electroporation of mouse zygotes.
(**a**) Electroporator NEPA21. (**b**) Glass chamber with metal plates. (**c**) Tweezer electrodes. (**d**) Glass micro-capillary pipette with silicone tube and 2.5 mL syringe. Scale bars represent 5 cm.

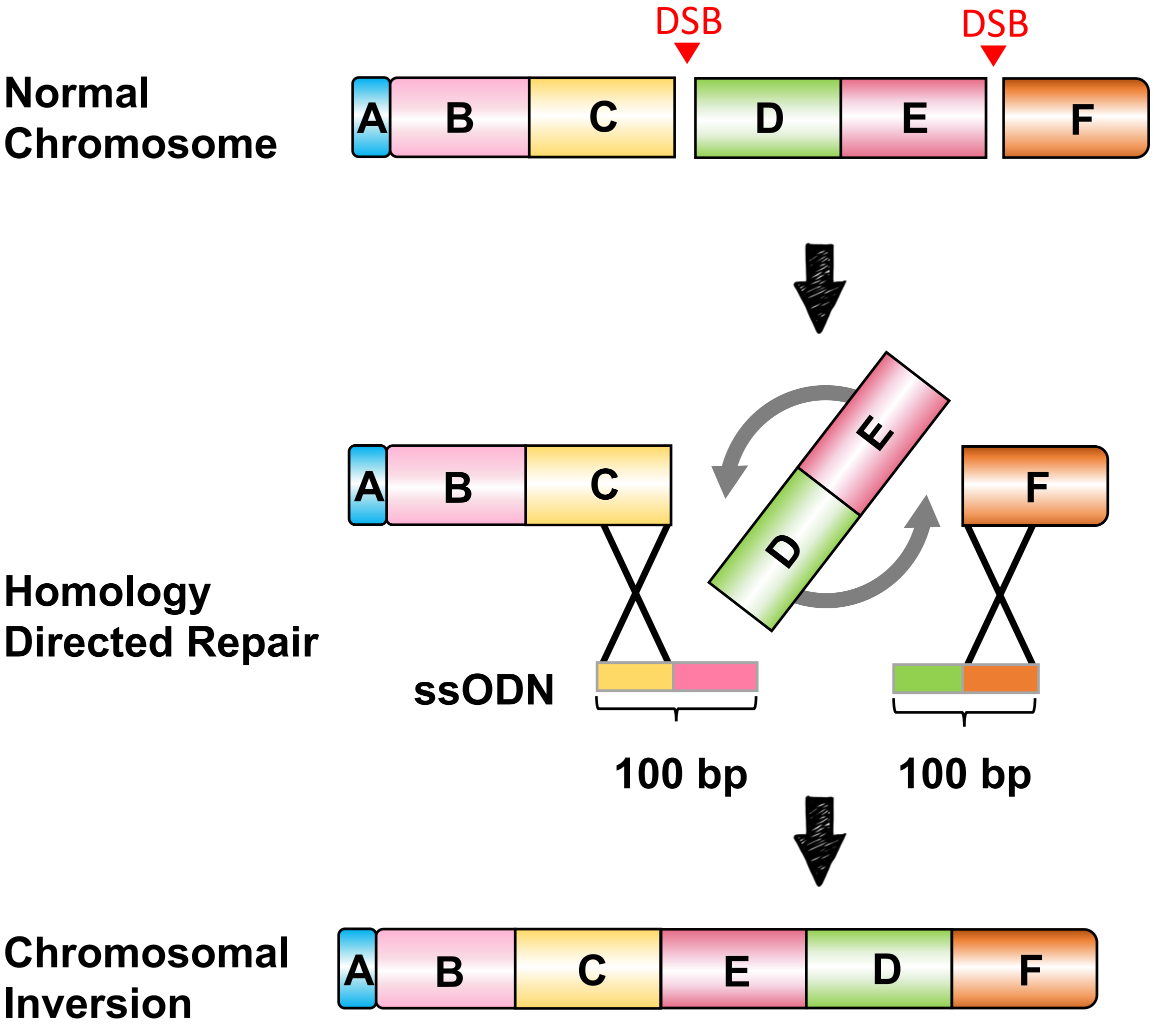

Supplementary Fig. 2: Schematic design for creating chromosomal inversion using the CRISPR/Cas9 system.
Double-strand breaks are repaired using HDR via the ssODNs or other mechanisms, leading to genomic rearrangements.

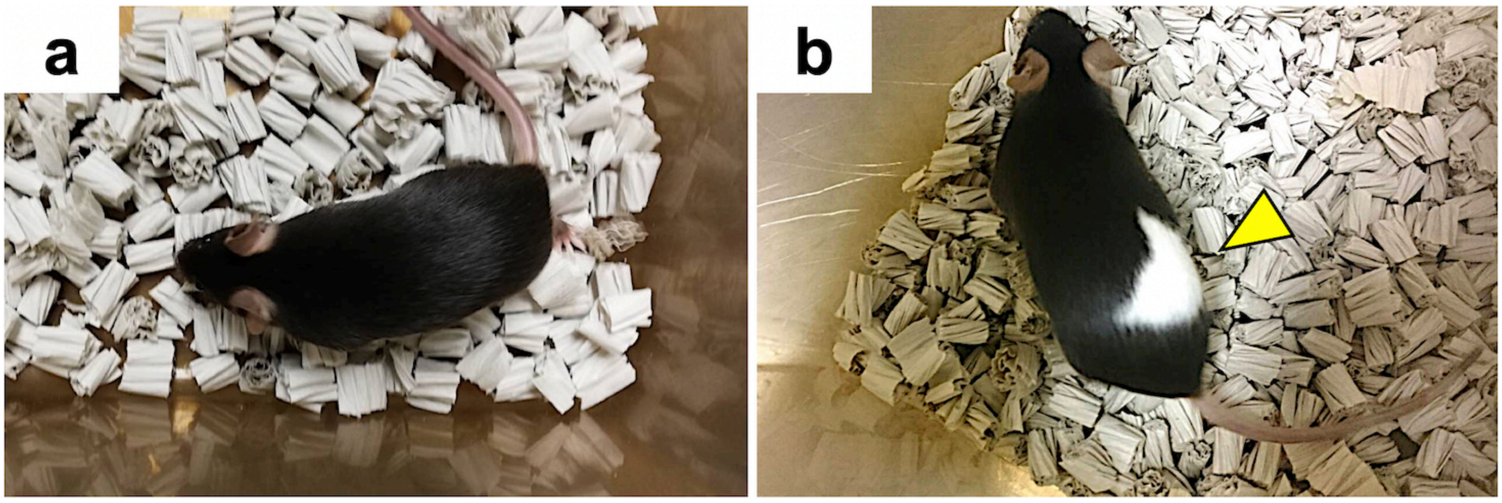

Supplementary Fig. 3: Production of the chromosome-rearranged mice via *in vitro* electroporation.
(**a**) Wild-type mouse. (**b**) Chromosome-rearranged mouse. The white spot on the mouse is indicated by a yellow arrowhead.

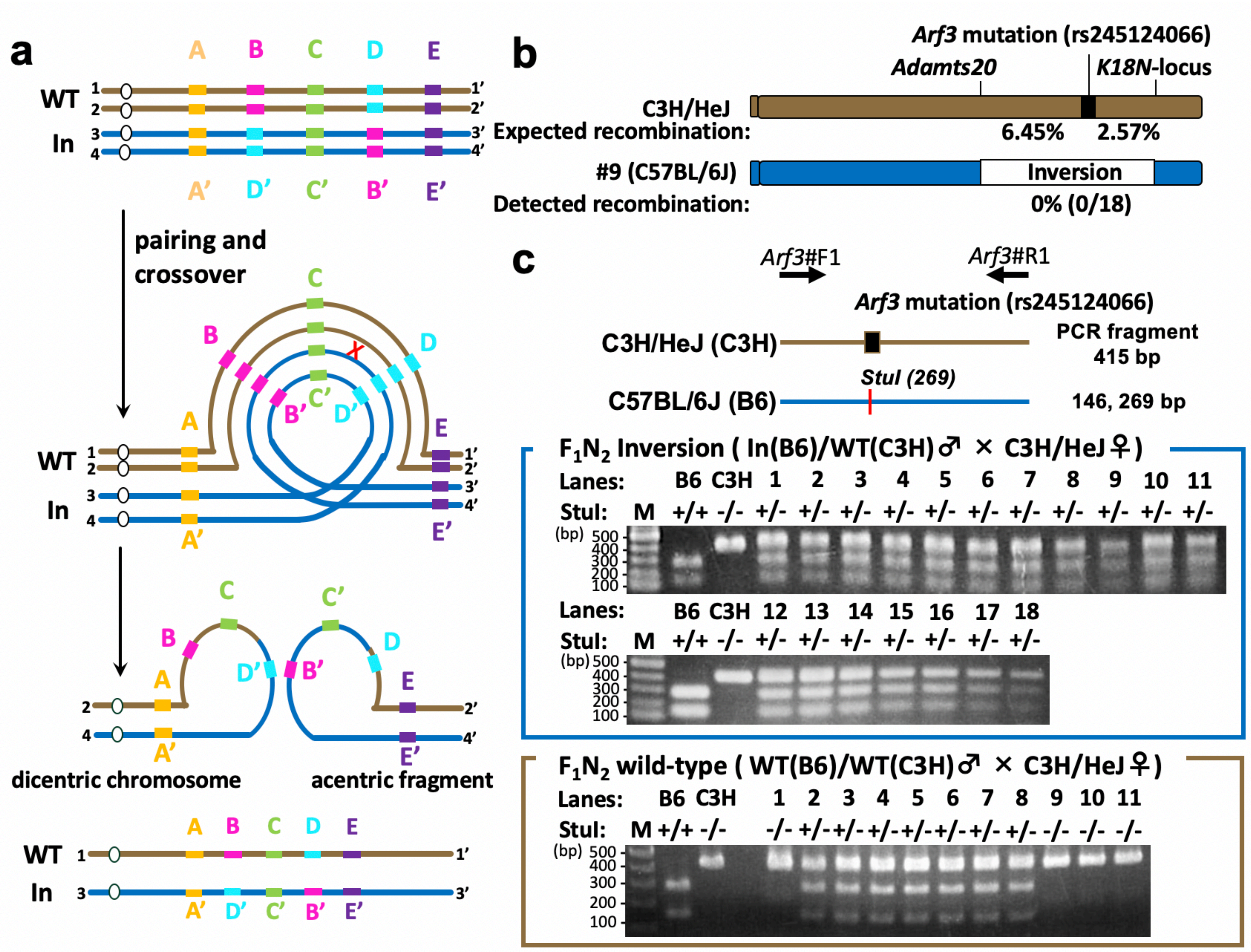

Supplementary Fig. 4: Chromosomal inversion #9 can suppress recombination.

(**a**) A single crossover between a wild-type (WT) chromosome and an inversion (In) leads to inviable dicentric and acentric products. (**b**) The recombination ratio between the *Admats20* and the K18N-locus. Recombination was suppressed by the inversion. (**c**) Detection of the *Arf3* mutation (rs245124066) via PCR-StuI digestion. The StuI restriction enzyme cuts the PCR products of the WT *Arf3* (+/+) and the heterozygous *Arf3* (+/-), but it does not cut the homozygous *Arf3* (-/-) PCR fragment. Genotyping was performed to determine the *Arf3* mutation.

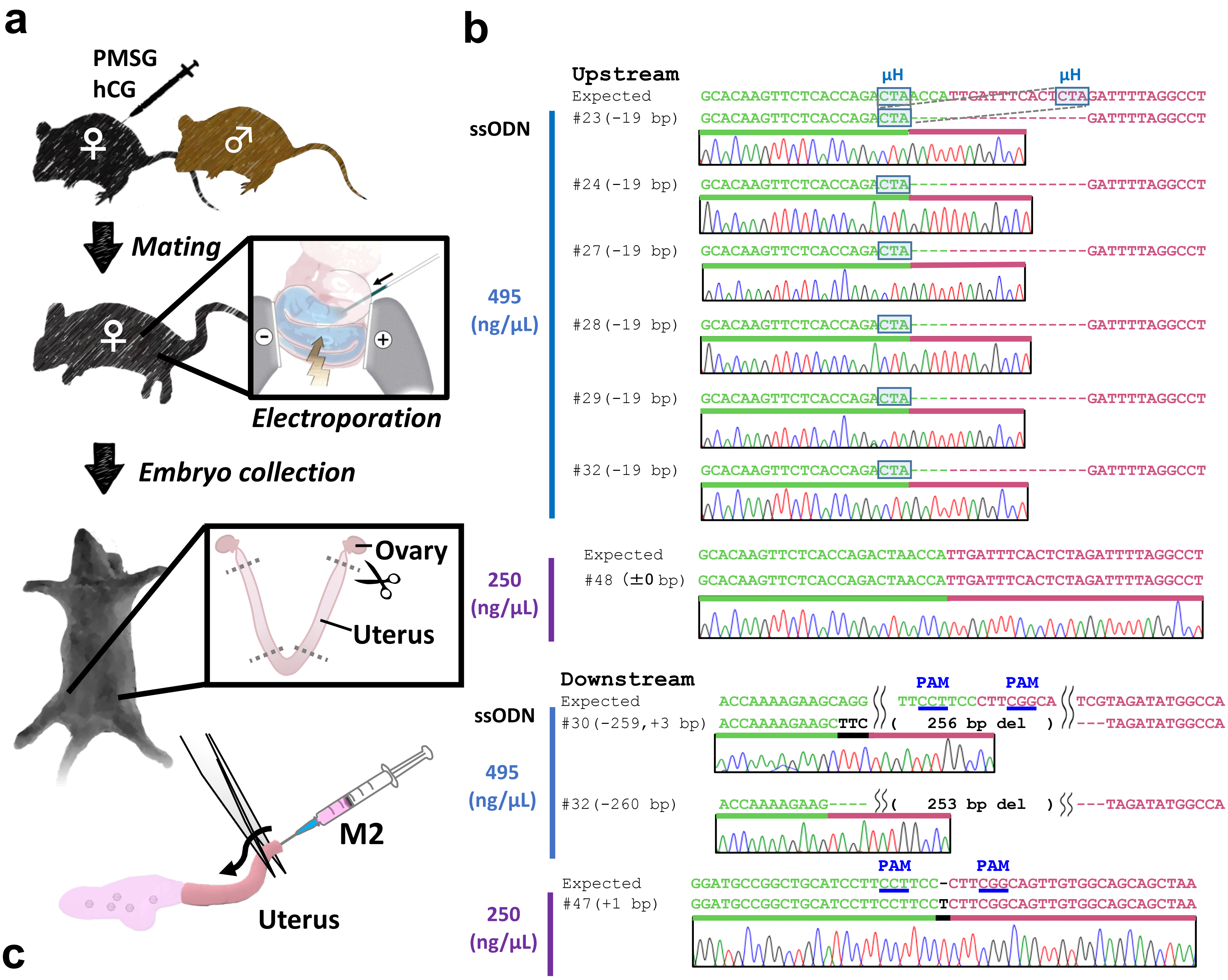

c

Cas9 targets	Distance (Mb)	ssODN (ng/μL)	Mice electroporated	Genotyped blastocysts	Inversion (%)	
					One breakpoint detected	Two breakpoints detected
PN-locus G30470N-locus	4.54	495	4	11	6 (54.5)	1 (9.1)
		250	4	16	2 (12.5)	0 (0)

Supplementary Fig. 5: Overview of blastocyst-stage embryo collection after *in vivo* electroporation and the sequences of the inversion junctions.
(**a**) Experimental procedures for blastocyst-stage embryo collection. Embryos are flushed from the uteri at 3 days after *in vivo* electroporation. (**b**) Alignment of sequences corresponding to the PN-locus and G30470N-locus genomic breakpoint junctions. The nucleotides highlighted in blue may work as the microhomology sequences for MMEJ. (**c**) Summary of the experimental efficiency of chromosomal inversion via *in vivo* electroporation.

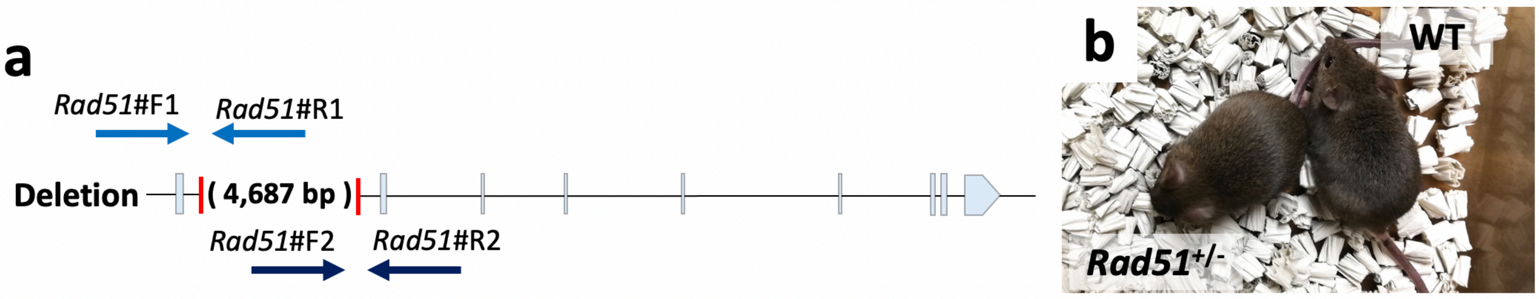

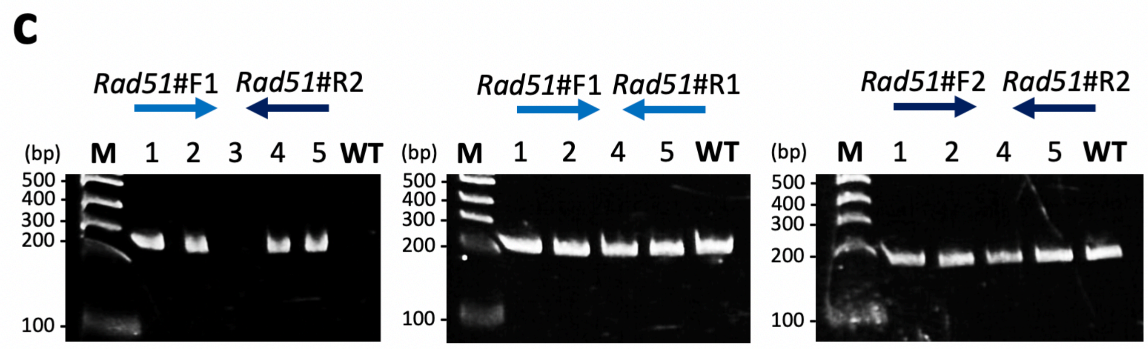

d

Genotype	No. observed
Wild-type	3
Heterozygous	15
Homozygous	0

Supplementary Fig. 6: Genotype distribution in offspring from intercrossed heterozygous *Rad51* mice.

(**a**) Positions of primers used for PCR screening of the *Rad51* deletion. (**b**) Wild-type mouse (WT) and heterozygous *Rad51* mouse (*Rad51*$^{+/-}$). *Rad51*$^{+/-}$ mice show no abnormality in outer appearance. (**c**) PCR amplification of the breakpoint junctions in wild-type mice (WT) and offspring from heterozygous *Rad51* intercrosses. (**d**) Summary of genotype distribution in offspring from heterozygous *Rad51* intercrosses.

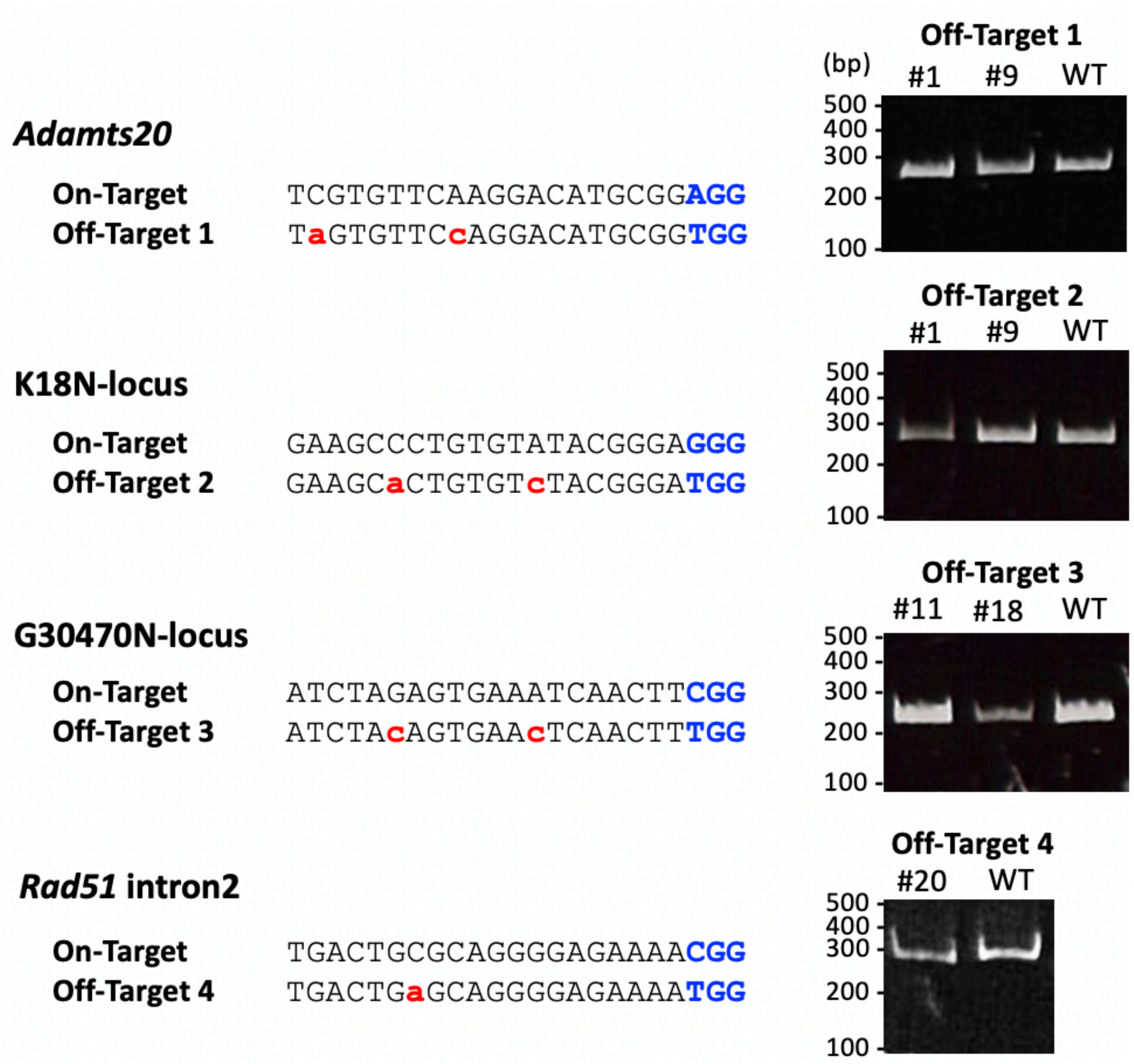

Supplementary Fig. 7: Off-target analysis by the T7E1 assay in the chromosome-rearranged mice.

Putative off-target locus PCR followed by T7E1 restriction was performed to detect off-target events. The PAM sequences and the mismatched nucleotides are in blue and red, respectively. No mutations were detected using the T7E1 assay at the four off-target sites.

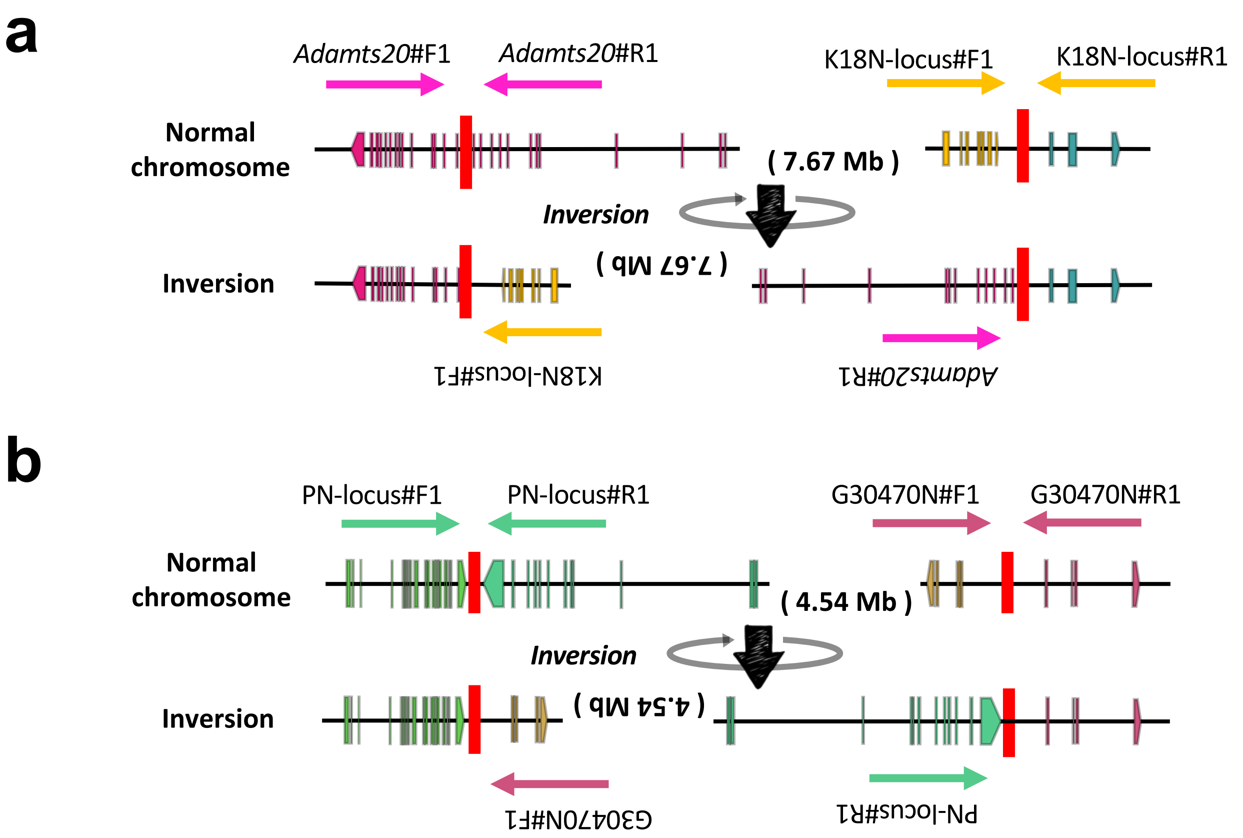

Supplementary Fig. 8: Schematic representation of the PCR primer positions for the screening of chromosomal rearrangements.

Positions of primers used for PCR screening of (**a**) the inversion between *Adamts20* and the K18N-locus and (**b**) the inversion between the PN-locus and G30470N-locus.

Supplementary Table 1: List of gRNAs used in the present study

			CHOPCHOP (https://chopchop.cbu.uib.no/)				
			Number of mismatches				
Target loci	Genomic location	Target Sequences (PAM)	0	1	2	3	Efficiency
Adamts20	Chr15: 94347708	TCGTGTTCAAGGACATGCGG (AGG)	0	0	1	3	71.44
K18N-locus	Chr15: 102034833	GAAGCCCTGTGTATACGGGA (GGG)	0	0	1	4	61.49
PN-locus	Chr11: 74673310	TCTCACCAGACTAACCAGGA (AGG)	0	0	0	7	69.80
G30470N-locus	Chr11: 79211879	ATCTAGAGTGAAATCAACTT (CGG)	0	0	1	12	54.49
Rad51 intron1	Chr2: 119113320	TCAGGTTCACTTCTGTGGTC (CGG)	0	0	4	11	47.60
Rad51 intron2	Chr2: 119118007	TGACTGCGCAGGGGAGAAAA (CGG)	0	1	4	16	29.76

Supplementary Table 2: List of ssODNs used in the present study

Target loci	Target Sequences (5'-3')
Adamts20::K18N-locus (Upstream)	CGTACTCGGGCCGGTCACAGAGCCGGGCTGTGCTCTTGATTCCACCTCCGGGAGGGGATAATCAGTCAGGTGCCTCGGCTCAGGTTTCTCGGGTCAGCTT
Adamts20::K18N-locus (Downstream)	GATGGCGAGTGGGGACCATGGGGACCCTACAGCTCGTGTTCAAGGACATGCGTATACACAGGGCTTCGCAGTTCCCAGGGCTCTTACGCATTTGATCCTC
PN-locus::G30470N-locus (Upstream)	GCAGAAAATTGCTGCTAGCAGTTGAATTCATGTCTGCACAAGTTCTCACCAGACTAACCATTGATTTCACTCTAGATTTTAGGCCTGTCAGACTGGAGGGAAGATGCTCCAGAGTTCATT
PN-locus::G30470N-locus (Downstream)	CCTCTGTCCTGAAGGCAGGGAGATAGCTCTGTGCAGGATGCCGGCTGCATCCTTCCTTCCCTTCGGCAGTTGTGGCAGCAGCTAACGCTCAGGCATTCACTCAACAAACACTTACTGAAT

Supplementary Table 3: List of all primers used in the present study

Target loci	Genomic location	Sequences (5'-3')
Genotyping primers		
Adamts20#F1	Chr15: 94347562-94347583	TCTCTGAAACTCGCAGACTGAC
Adamts20#R1	Chr15: 94347823-94347844	TTCCTGTGTGTGTGCTTCTTCT
K18N-locus#F1	Chr15: 102034680-102034700	GGCATCAAATGTGTCTTCTCA
K18N-locus#R1	Chr15: 102034945-102034966	TGATGTCTGTGGCCTTTACTGT
PN-locus#F1	Chr11: 74673204-74673225	TTAGTATGTCTTGAGGTGGGGG
PN-locus#R1	Chr11: 74673662-74673683	GACTCAATTCCAGAGATGAGGG
G30470N#F1	Chr11: 79211712-79211733	TTCCTCACAGCAACAGAACAGT
G30470N#R1	Chr11: 79212136-79212157	TCATCTGAAAGTTGGGTGTCAG
Rad51#F1	Chr2: 119113213-119113234	ACCGACCTGTACTTCAAGCATT
Rad51#R1	Chr2: 119113422-119113443	ACCAGCCACTACCCTCATTTTA
Rad51#F2	Chr2: 119117961-119117982	TCCCATTTCTTCTTTGAGTGGT
Rad51#R2	Chr2: 119118134-119118155	ACTTGCATTCTCTGGCTTTCTC
Arf3#F1	Chr15: 98767189-98767210	TGGTACACAAGCATACATGCAG
Arf3#R1	Chr15: 98767582-98767603	CTGTGACCAAAAACAAGCTGAG
PN-locus_1st#F1	Chr11: 74673204-74673225	TTAGTATGTCTTGAGGTGGGGG
PN-locus_1st#R1	Chr11: 74673662-74673683	GACTCAATTCCAGAGATGAGGG
PN-locus_2nd#F2	Chr11: 74673257-74673278	TGACTCAGAAGCAGAAAATTGC
PN-locus_2nd#R2	Chr11: 74673505-74673526	GTGTCTTCCCAACCAAAAGAAG
G30470N_1st#F1	Chr11: 79211712-79211733	TTCCTCACAGCAACAGAACAGT
G30470N_1st#R1	Chr11: 79211979-79211999	CTTCTGTGCATGGCCATATCT
G30470N_2nd#F2	Chr11: 79211712-79211733	TTCCTCACAGCAACAGAACAGT
G30470N_2nd#R2	Chr11: 79211979-79211999	CTTCTGTGCATGGCCATATCT
DNA probe primers		
Adamts20(15qE3)#F1	Chr15: 94321710-94321744	ACCAGTAAGGCTTATGGCATATGAAAACAGCCAGC
Adamts20(15qE3)#R1	Chr15: 94331513-94331547	ATTACCATCTCCTCTCTAGGTGGCACATCATTGGG
K18N(15qF2)#F1	Chr15: 102008124-102008158	AAGATGCATAGTTGATGGGAAGGACTGTGTCATCG
K18N(15qF2)#R1	Chr15: 102021343-102021377	CAATTCTCCTGCCTCAGTCTCTCAGGCACTACAAG
G41405N(15qF2)#F1	Chr15: 102040214-102040248	ATGCACTGGTACACATGCAGGCAAAATACTCATGC
G41405N(15qF2)#R1	Chr15: 102050559-102050593	ACTCTAGCCCAAGAGGGGCCTGAACTCACTACATA
T7E1 assay primers		
OTS1#F1	Chr7: 82556869 -82556889	AGGAATGCAGAGAGGAAAAGC
OTS1#R1	Chr7: 82557120 -82557141	ATTCAAAAAGCTGCCATCAGTT
OTS2#F1	Chr9: 1108448098-108448119	ATAGGTGTGAGCATGTGTGTCC
OTS2#R1	Chr9: 108448344-108448365	GACTGTTTGTTATTGGGGGTGT
OTS3#F1	Chr14: 100921155-100921179	CACAACTTTTGAAAATTATAAGGGA
OTS3#R1	Chr14: 100921346-100921367	GCTTTCATGATTGTGGCATAGA
OTS4#F1	Chr8: 54383489-54383511	GATGAACTTGAAGGGCATTTTC
OTS4#R1	Chr8: 54383755-54383777	TTCTGCTTCCTTTTTATCCTGC
